# Artificial modulation of cell width significantly affects the division time of *Escherichia coli*

**DOI:** 10.1038/s41598-020-74778-3

**Published:** 2020-10-20

**Authors:** Baihui Liang, Baogang Quan, Junjie Li, Chantal Loton, Marie-Florence Bredeche, Ariel B. Lindner, Luping Xu

**Affiliations:** 1 Center for Nano and Micro Mechanics, School of Aerospace Engineering, Tsinghua University, Beijing, 100084 People’s Republic of China; 2 Beijing National Laboratory for Condensed Matter Physics, Institute of Physics, Chinese Academy of Sciences, Beijing, 100190 People’s Republic of China; 3 School of Physical Sciences, University of Chinese Academy of Sciences, Beijing, 100049 People’s Republic of China; 4 Songshan Lake Materials Laboratory, Dongguan, 523808 Guangdong People’s Republic of China; 5 Systems Engineering and Evolution Dynamics Lab, INSERM U1001, Paris Descartes University, 75014 Paris, France; 6 Faculty of Medicine, Paris Descartes University, 75014 Paris, France; 7 Centre for Research and Interdisciplinarity (CRI), Paris Descartes University, 75014 Paris, France

**Keywords:** Biophysics, Biotechnology, Microbiology

## Abstract

Bacterial cells have characteristic spatial and temporal scales. For instance, *Escherichia coli*, the typical rod-shaped bacteria, always maintains a relatively constant cell width and cell division time. However, whether the external physical perturbation of cell width has an impact on cell division time remains largely unexplored. In this work, we developed two microchannel chips, namely straight channels and ‘necked’ channels, to precisely regulate the width of *E. coli* cells and to investigate the correlation between cell width and division time of the cells. Our results show that, in the straight channels, the wide cells divide much slower than narrow cells. In the ‘necked’ channels, the cell division is remarkably promoted compared to that in straight channels with the same width. Besides, fluorescence time-lapse microscopy imaging of FtsZ dynamics shows that the cell pre-constriction time is more sensitive to cell width perturbation than cell constriction time. Finally, we revealed a significant anticorrelation between the death rate and the division rate of cell populations with different widths. Our work provides new insights into the correlation between the geometrical property and division time of *E. coli* cells and sheds new light on the future study of spatial–temporal correlation in cell physiology.

## Introduction

Bacterial cells usually have conservative shapes, sizes^1^, and division time^2^. The combination of these spatial and temporal properties has evolved in coordination to maximize the survivability and reproduction efficiency of the species^3,4^. Three kinds of models have been proposed to explain how bacterial cells control their size homeostasis throughout the cell cycle^5^. The first one is the ‘timer’ model, in which bacterial cells would grow for a specific amount of time between two successive divisions^6^. The second one is the ‘sizer’ model, in which cells commit to division on reaching a size threshold^7^. The third is the well-known ‘adder’ model, in which bacterial cells achieve their size homeostasis by adding a constant length^8^, volume^9^, surface area^10^, or ‘adder-per-origin’^11^ before division, irrespective of cell size at birth. However, these pioneering works postulate that cells grow and divide under the natural condition without any size perturbation. For instance, *Escherichia coli*, the rod-shaped bacteria always grow along the long axis of the cell and maintain its cell width constant during the cell cycle. However, whether those models still work out when their cell width is disturbed, remains to be explored.

In recent years, much progress has been made in understanding the *E. coli* cellular physiology when its cell geometry is artificially disturbed. These perturbations can be achieved through various biological, chemical, and physical methods, such as cytoskeletal mutation^12,13^, chemical treatment with A22 (S-3,4-dichlorobenzyl-isothiourea)^14,15^, or external physical constraints^16,17^. Männik et al. squeezed *E. coli* cells into irregular shapes by narrow silicon channels and showed that cells manage to divide into two equal-sized daughter cells regardless of their abnormal shapes^18^. Wu et al. studied the Min oscillation pattern of *E. coli* with large size and diverse geometric shapes using A22 and cephalexin combined with agarose microchambers^19^. These experiments have revealed crucial roles that *E. coli* cell shape and size play in cellular physiology; however, it is still largely unknown whether the perturbation of cell width will significantly affect their division time.

Notably, bacterial cell division is a complex process that contains numerous molecular events, including chromosome replication and segregation^20^, division site positioning^21^, septum assembly^22^, cell constriction coupled with cell wall synthesis^23^, some of which might be sensitive to cell width. For instance, the septum assembly and cell constriction of *E. coli* are facilitated by cell divisome, a dynamic multiprotein assembly localizing at mid-cell to synthesize new peptidoglycan and to constrict cell envelope^24^. In *E. coli*, the cell divisome consists of at least 34 different proteins^25^, where FtsZ is required for cytokinesis, forming a ring-shaped structure that treadmills along the cellular circumference, mediated by membrane-attached proteins FtsA and ZipA^26,27^. This ‘Z-ring’ recruits other division proteins and generates contractile force for cell envelope constriction^28^. Previous researches show that Z-ring positioning is robust against the fluctuation of cell width^29^. However, other cell division related processes, such as Min oscillation^19,30^, chromosome replication^31,32^ and FtsZ turnover and polymerization dynamics^33^ are also subjected to the modulation of cell width. Despite these previous works, it remains largely unclear whether perturbation of cell width will affect cell division time of *E. coli*, due to the lack of proper experimental methods to investigate the division time of cells with different width systematically.

To address this issue, we developed two easy-to-use microchannel chips that enable us to monitor the correlation between cell width and cell division time of *E. coli* at the single-cell level. Using the chip of straight channels with various widths (0.8–2.8 μm), we found that there is a significant positive correlation between individual cell division time and its width. We then asked whether local constrains on cell width can lead to a significant effect on cell division time as well. To obtain local constraints on cell width, we developed microchannels with fixed width and local constriction regions along the channels. We discovered that, compared to the straight channels, the channels with the same width and local constriction lead the *E. coli* cells to much shorter division time. We then used fluorescence time-lapse microscopy to track the FtsZ dynamics and found that the cell width perturbation has a major impact on the time duration of both pre-constriction and constriction phases of the cell cycle, and the impact is more significant on the former one than the second. Finally, we discovered a remarkable anticorrelation between the death rate and the division rate of the cell population with various cell widths. Our work, for the first time, revealed how physical modulation of cell width leads to the significant change of cell division time and survivability of *E. coli*, which will shed new light on further exploration of the interplay between cell morphology and physiology.

## Results

### Sculpting living cells with defined widths

Previous studies have introduced various microfluidic and micropattern techniques to explore cell physiology with arbitrary morphology^18,29^. For instance, the microfluidic ‘mother machine’ system has been developed to enable long-term observation of cell growth in a chemostat environment with single-cell resolution^34^. However, if cells with large size and deformability enter the channel of mother machine, the diffusion of nutrition will be largely blocked by the cells closed to the entry, which will lead the other cells in the channel to a severe lack of nutrition (Supplementary Fig. S1a). The agarose microchambers, on the other side, can provide homogenous nutrition supply despite the aberrant cell morphology^19,35^. However, after a period of growth and division, the cells grow, divide, squeeze the microchambers, and eventually cause the microstructure to deform or collapse, which makes it difficult to obtain high-quality imaging of the growth and division of cells in long term (Supplementary Fig. S1b).

**Figure 1:**
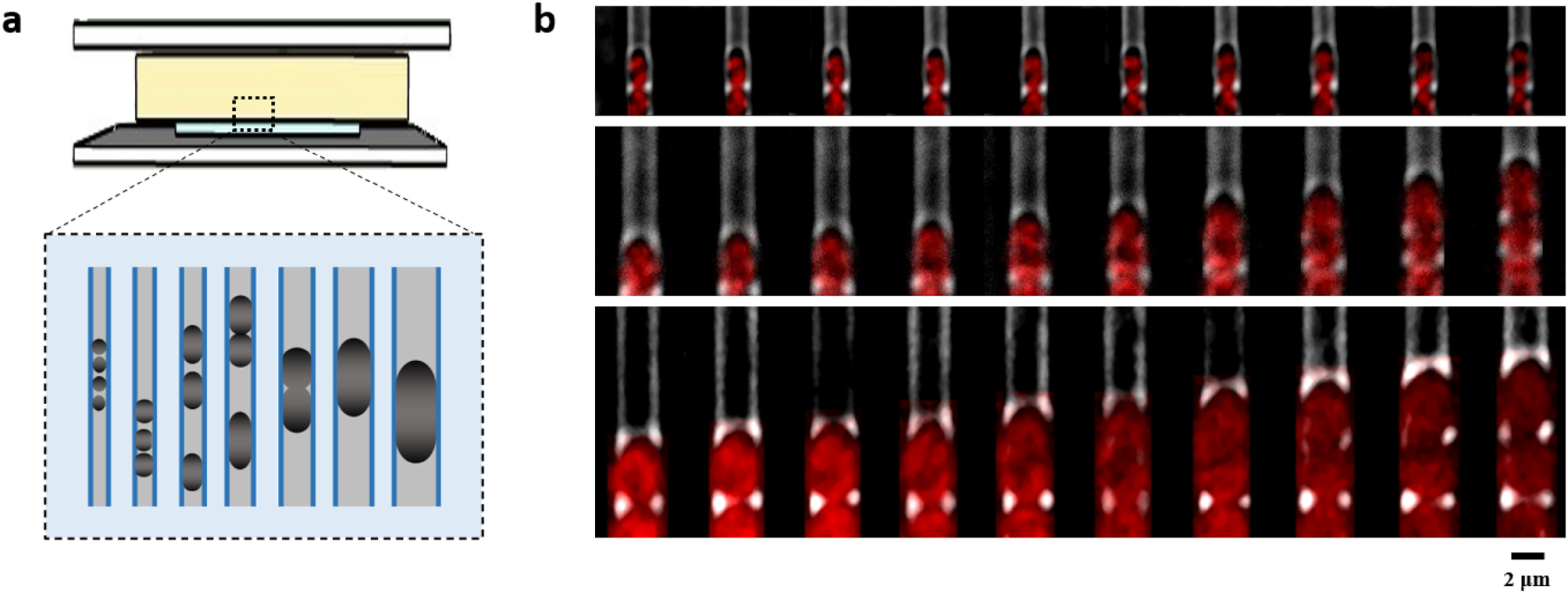
Sculpting living cells with defined widths. (**a**) Schematic of the ‘sandwich’ microchannel chip. The chip is composed of two coverslips (top and bottom: light gray), an agarose pad supplemented with nutrients (middle: light yellow), and a PDMS layer with microchannels (middle: light blue). The enlarged section is the top view of microchannels with various widths. (**b**) Bright and fluorescence field merged time-lapse images of three representative cells growing in microchannels with specific widths, HU-mCherry labeled nucleoid (red). From top to bottom, the widths of microchannel are 1.4 μm, 2.0 μm, and 2.6 μm, respectively. The time intervals for three images series are 2 min, 4 min, and 4 min, respectively.

In this study, we developed an easy-to-use ‘sandwich’ microchannel chip, to precisely control the widths of *E. coli* cells and obtain high-quality and long-term cell division imaging. This microchannel chip consists of an agarose pad layer, a thin PDMS layer with microchannels, and two coverslips (Fig. 1a). The agarose pad is used to supply nutrients containing the Luria–Bertani medium with A22, an antibiotic that antagonizes the dynamics of bacterial cytoskeleton protein MreB, which facilitates the deformation of cells. The microchannels in the PDMS layer are 1 μm deep, 60 μm long, and with various widths ranging from 0.8 to 2.8 μm, applied to sculpture the morphology of cells with determined width. The coverslips on the top and bottom prevent the drying of the agarose layer and offer the support of the sandwich structure. Due to the function of A22, *E. coli* cells seeded in the microchannels gradually grow into a round shape and eventually adapt to the border of the channels. With their widths limited by the channels, cells grow and divide along the long axis of the channels. We then take time-lapse images of cells living in the channels every two minutes for two hours (Fig. 1b). Although the deformed *E. coli* cells in our experiment are one to ten times larger in volume than wild type cells, most of them manage to divide around the volumetric center, which indicates the remarkable robustness of cell division site determination even with such significant perturbation of cell size in our work.

### Wide cells divide much slower than narrow cells

**Figure 2:**
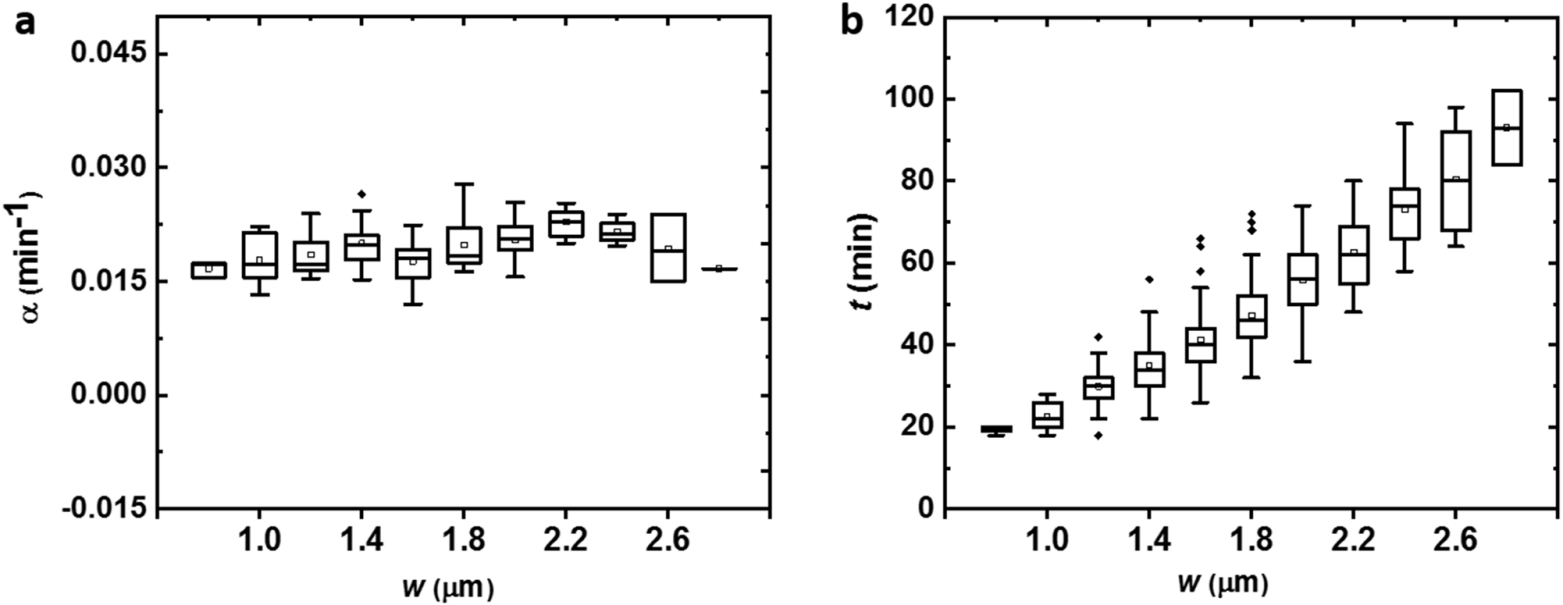
Cell biomass growth rate α and division time *t* of cells with different widths. (**a**) The box chart presenting cells growing in channels with different widths *w* have a uniform growth rate α. (**b**) Cell division time *t* increases as cell width *w* increases. see Supplementary Table S3 for sample size per width.

We then investigated whether and how cell width perturbation influences cell growth and division time of *E. coli*. Since the depth of the channel is fixed, the measurement of cell biomass growth rate is equivalent to measuring the growth rate of its cell area by a series of time-lapse images. Our data shows that, despite their widths, all the cells in microchannels follow exponential growth law: *S*_*T*_ = *S*_*0*_* e*^*αT*^, where *S*_0_ and *S*_*T*_ indicate the cell area at the initial time *T*_0_ and the time *T*, respectively, and *α* indicates the biomass growth rate for an individual cell. The statistical result shows that cells exhibit a relatively uniform growth rate in biomass despite the variation of initial cell area of cells with different widths (*α* = 0.019 ± 0.004 min^−1^, Mean ± SD, *P* > 0.05, Fig. 2a and Supplementary Fig. S2). Previous research shows that severe mechanical deformation could lead to slower growth^18^. However, our results indicate that the biomass growth rate of *E. coli* is robust to the change of cell width by this gentle physical constrains.

Given the growth rate of biomass maintains undisturbed when cell width changes, we then explored whether cell division time *t* is also robust to the change of cell width *w*. To this end, we applied a threshold method to determine the initiation and accomplishment of the cell division process and calculate the cell division time as the time duration between two successive cell division events (Supplementary Fig. S3). The results of the division time of cells growing in channels with different widths show that there is a significant positive correlation between cell division time *t* and cell width *w* (Pearson’s *r* = 0.98; Fig. 2b). Generally, wide cells divide much slower than narrow cells. For instance, those cells at 0.8 μm spend less than 20 min for one division, while those at 2.8 μm have an average division time of 93 min, which is about 4.7 times of the former one. It is also worth noting that wide cells have a larger variation of division time than that of narrow cells (CV_0.8 μm_ = 7.8%; CV_2.8 μm_ = 14%), see in Supplementary Table S3. The discovery of the constant biomass growth rate and positively correlated cell division time with the width micro-channels offers a straightforward explanation to the observation that *E. coli* cell size at the end of cell division (*S*_*E*_) increases with the width of channels (see “Methods” and Supplementary Fig. S4a).

### Local constriction of the cell width helps accelerate the process of cell division

**Figure 3:**
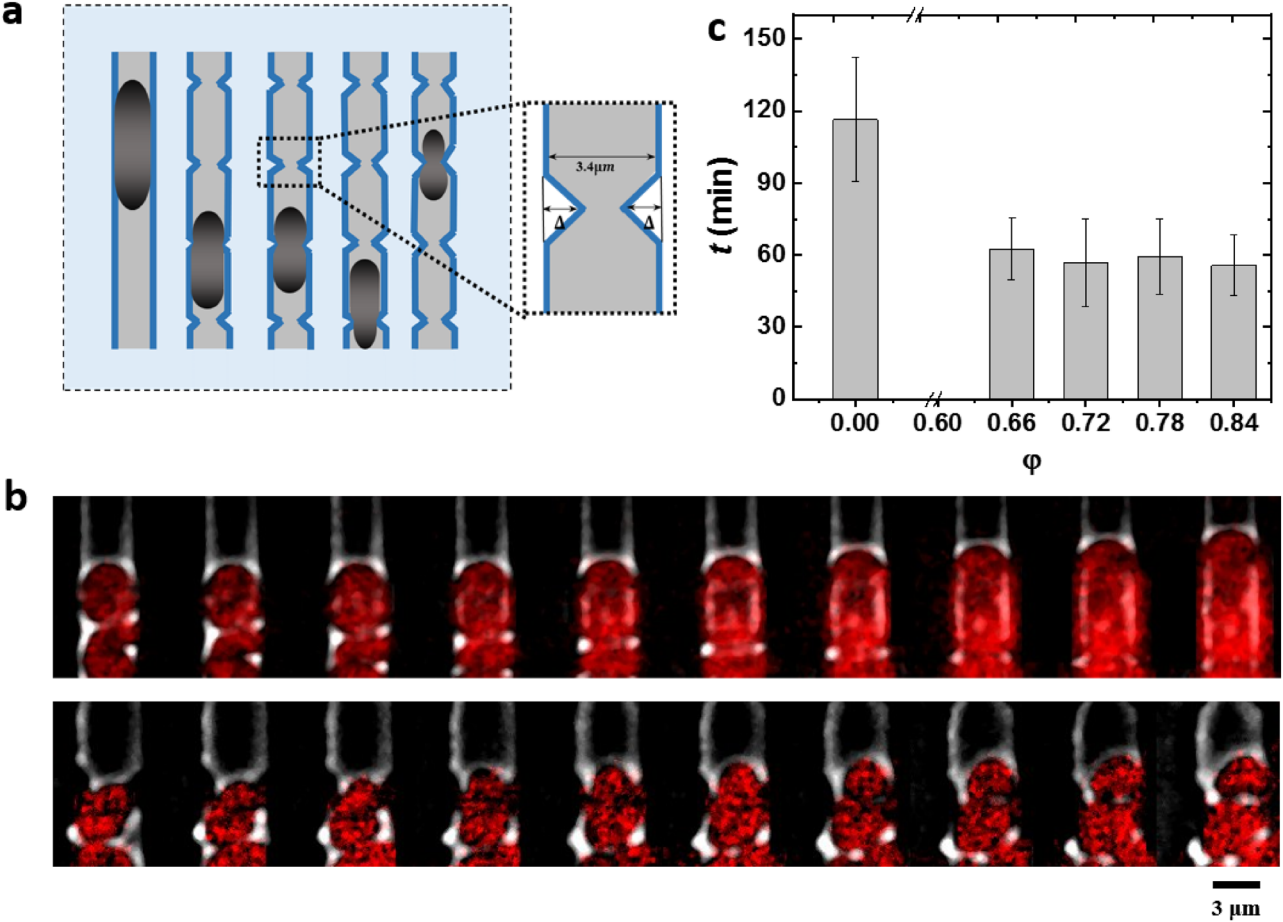
Cell division is promoted in ‘necked’ channels. (**a**) Schematic of microchannels with periodic necks. The enlarged section is the top view of the necks. (**b**) Time-lapse images of two representative cells with fluorescently labeled nucleoid (HU-mCherry), which are in straight channel (upper panel, $\varphi$ = 0, time interval is 4 min) and ‘necked’ channel (lower panel, $\varphi$ = 0.84, time interval is 2 min). (**c**) The correlation between division time *t* and the constriction ratio $\varphi$ for cells in ‘necked’ channel. For all quantitative data, each value is mean ± SD. Sample size for each group is shown in Supplementary Table S4.

As we have shown that changing the width of entire cells of *E. coli* will result in a significant change of cell division time, it is of great interest to ask whether this effect could be local, that is, whether local modification of cell width has an impact on cell division time? To answer this question, we developed a dedicated sandwich chip with ‘necked’ channels: all ‘necked’ channels with fixed width of 3.4 μm and periodic local constrictions (referred as ‘necks’). We define $\varphi$ = 2∆/*w* as the constriction ratio, where $\Delta$ and *w* represent the half-constriction distance and the width of the channel, respectively (Fig. 3a). We applied fluorescence time-lapse microscopy to follow and compare the growth and division of cells in the straight and ‘necked’ channels (Fig. 3b). We firstly investigated whether local regulation of cell width affects the biomass growth rate of *E. coli.* Our results show that cells in ‘necked’ channels also have a uniform growth rate (*α* = 0.018 ± 0.003 min^−1^, Mean ± SD, *P* > 0.05, Supplementary Fig. S2b), which is in line with the result of cells in straight channels (*P* > 0.05). This indicates that the local constriction of cell width has no obvious effect on the biomass growth of cells.

We then ask whether these necks influence on cell division time. Thus, we compared the division time *t* of those cells in the ‘necked’ channels that were locally restricted by the necks throughout their cell division process with the cells in neck-free straight channels of the same width. In this work, only those cells who managed to accomplish at least one full cell division in our experimental time are considered. The result shows that the ‘necked’ cells divide significantly faster than those in straight channels (****P < 0.0001; Fig. 3c). Quantitatively speaking, cell division time in straight channels (116.3 ± 25.8 min, Mean ± SD, $\varphi$ = 0) is nearly twice larger than the ‘necked’ ones (55.7 ± 12.7 min, Mean ± SD, $\varphi$ = 0.66). This result indicates that local physical constriction indeed has a significant impact on the cell division time of *E. coli*.

### The pre-constriction phase of cell division process is more sensitive to cell width perturbation

**Figure 4:**
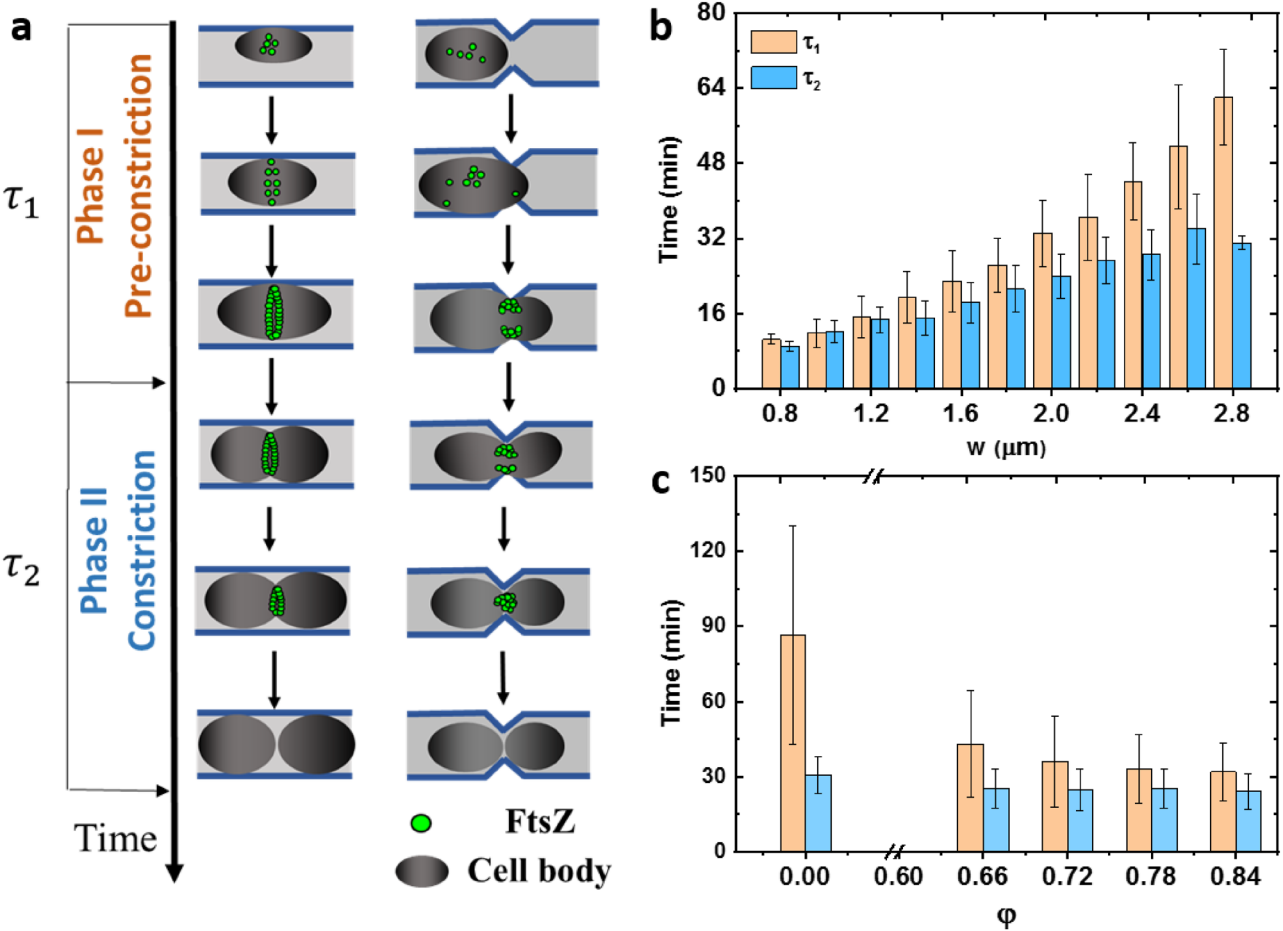
Width perturbation affects the time spent on two phases of the cell division process. (**a**) The criterion to determine two phases for both straight and ‘necked’ channels is shown. The cell division process is divided into two phases, namely the pre-constriction phase (phase I) and the constriction phase (phase II). (**b**) For cells growing in straight channels, both *τ*_1_ and *τ*_2_ increase as cell width *w* increases. Each value is Mean ± SD. (**c**) For cells growing in ‘necked’ channels, *τ*_1_ decreases significantly compared to *τ*_2_. Each value is Mean ± SD.

Our work has shown that cell division time can be largely affected by the width regulation of the whole cell or just on the local regions of the cells. Then, given the complicated processes related to the cell division, we try to determine which stages of the cell division process are sensitive to the perturbation of cell width. Previous research shows that the time spent on the ‘D’ period of the cell cycle increases with the increasing cell width^11^. A straightforward explanation is that, given that the Z-ring constriction is rather constant under the same growth condition^33^, the increase in cell width leads to larger circumference and an increase in cell constriction time. However, whether this is the only or major route for cell width to influence division time is to be explored. To precisely quantify the impact of cell width on cell division machinery, we constructed a fusion fluorescent protein FtsZ-gfpmut2 as a marker molecule to track the dynamics of FtsZ molecules and Z-ring during the whole cell cycle. During cell division process, FtsZ molecules undergo a multi-step process, including dispersion in cytoplasm, aggregation on cell membrane, formation of Z-ring, constriction of Z-ring and eventually dissociation (Supplementary Fig. S5a). We divide the whole cell division process accordingly into two major phases, namely the pre-constriction phase (phase I) and the constriction phase (phase II). Here, phase I begins with the end of the last division and ends up forming a matured divisome (Z-ring or Z-arc), and phase II begins with the inward constriction of the divisome and ends with the dissociation of the FtsZ clusters eventually^36^. The criterion to determine the two phases for both straight and ‘necked’ channels is illustrated in Fig. 4a and Supplementary Fig. S5c. *τ*_1_ and *τ*_2_ denote the time duration of phase I and phase II, respectively.

We then apply fluorescence time-lapse microscopy to investigate the strength of the impact of cell width on *τ*_1_ and *τ*_2_. In straight channels, we find that although both *τ*_1_ and *τ*_2_ are increasing with cell width. *τ*_1_ increases much faster than *τ*_2_ (**P < 0.01, Fig. 4b). The ratio of time spent on both phases *ε*, i.e., *ε* = *τ*_1_/*τ*_2_, ranging from 1.1 to 2.0 as the cell width increases (Supplementary Fig. S6). On the other hand, in the ‘necked’ channels, *τ*_1_ is reduced significantly compared to cells in straight channels (****P < 0.0001). In the meanwhile, *τ*_2_ remains relatively constant (*P* > 0.05, 25.6 ± 7.9 min, Mean ± SD, Fig. 4c). When constriction ratio $\varphi$ increases to 0.66, *ε* decreases significantly from 2.8 to 1.7 (Supplementary Fig. S7).

### Cell width perturbation has a significant impact on the population death rate

**Figure 5:**
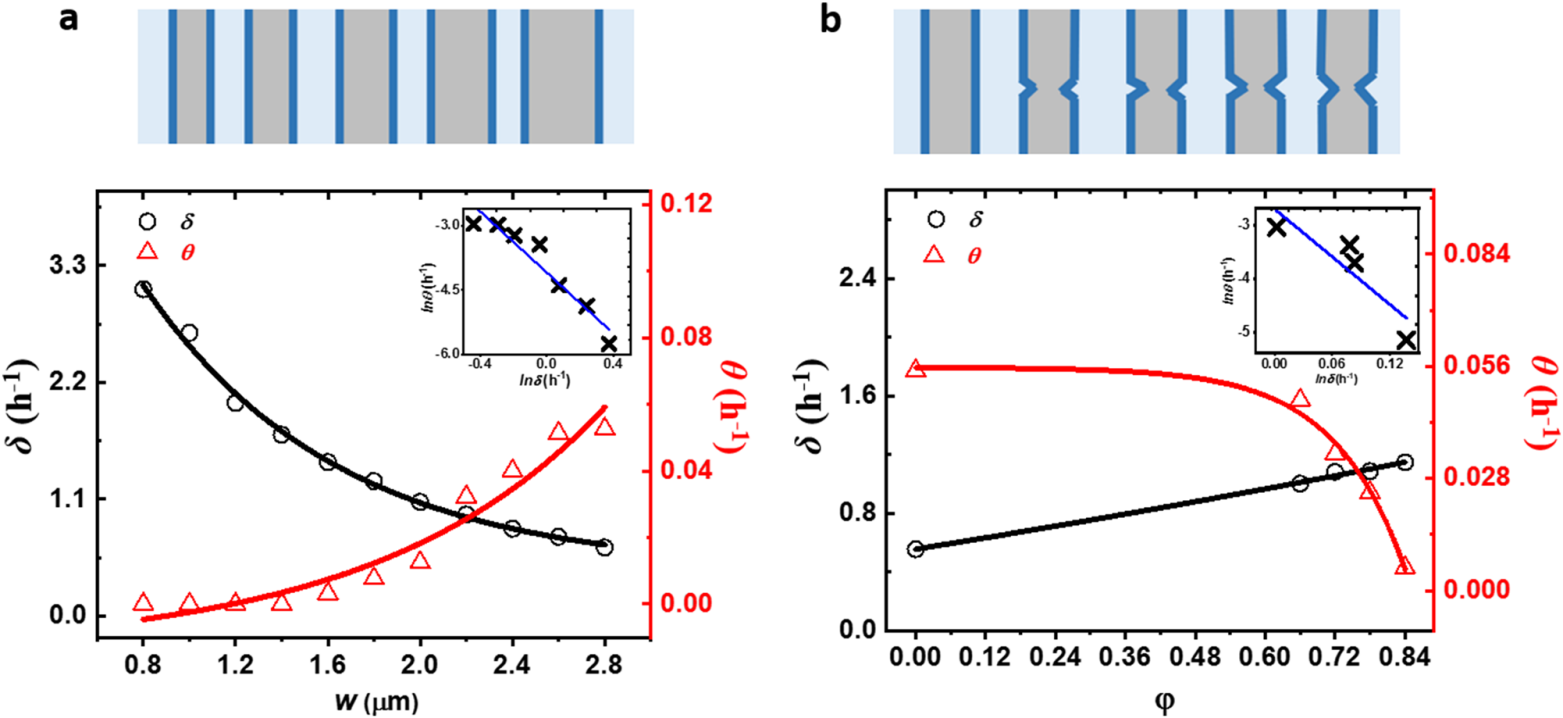
Cell width regulation affects the population division rate *δ* and death rate *θ*. (**a**) The population division rate *δ* and death rate *θ* for cells growing in straight channels. The black and red solid lines refer to the exponential fits of *δ* (*R*^2^ = 0.99) and *θ* (*R*^2^ = 0.93). The inset is the correlation between *lnθ* and *lnδ*. (**b**) The population division rate *δ* and death rate *θ* for cells growing in ‘necked’ channels. The black and red solid lines refer to the exponential fits of *δ* (*R*^2^ = 0.98) and *θ* (*R*^2^ = 0.97). The inset is the correlation between *lnθ* and *lnδ*.

In our experiment, we observed that some cells die, whose nucleoid fluorescence dissipate during the experimental period (Supplementary Fig. S8). It is of great interest to ask whether the perturbation of cell width affects not only division time but also survivability of cells. And if so, is there a correlation between population death rate and division rate when cell width is variable? To answer these questions, we measure the death rate *θ* of cell population and their according division rate *δ* with various cell width *w*. The death rate *θ* is defined as *θ* =  *m/NT*, where *N* is the total cell number and *m* is the number of cells that die during a period of *T*. The division rate *δ* is defined as *δ* = 1*/t,* where *t* is the division time of individual cell. In straight channels, we find that when cell width changes from 0.8 μm to 2.8 μm, the division rate *δ* decreases from 3 to 0.6 h^−1^, the death rate *θ* increases from 0 to 5.3 $\times$ 10^–2^ h^−1^ (Fig. 5a). In ‘necked’ channels, when $\varphi$ increases to 0.84, the death rate *θ* decreases from 5 $\times$ 10^–2^ to 0.6 $\times$ 10^–2^ h^−1^, while the division rate *δ* increases from 0.55 to 1.14 h^−1^ (Fig. 5b). To further explore the correlation between *θ* and *δ*, we plot *lnθ* versus *lnδ* for both straight and ‘necked’ channels. Result shows that there is a significant anticorrelation between the death rate and the division rate of the cell population both for straight channels (Person’s *r* = − 0.96, Fig. 5a inset) and ‘necked’ channels (Person’s *r* = − 0.89, Fig. 5b inset).

## Discussion

To summarize, in this work we investigate the correlation between cell width and division time of *E. coli* as a model system to reveal the crucial impact of geometrical properties on essential physiology of bacterial cells. By developing easy-to-use sandwich microchannel chips, we achieved both precise regulation of the width of the whole cell and local regions of the cell. Using the microchannel chips and fluorescence microscopy we found that the division time of *E. coli* is significantly correlated with cell width. The discovery that local necks can also modulate cell division rate indicates that local constriction of cell width is enough to influence cell division time. Besides, by tracking the dynamics of fluorescent FtsZ proteins, we found that the perturbation on cell width has a greater effect on pre-constriction phase. Finally, we revealed a significant anticorrelation between the death rate and the division rate of the cells with different widths. To the best of our knowledge, our work is the first systematic study on the correlation between cell width and cell division time, which will help future exploration of spatial–temporal interplay in cell physiology.

Proper spatial localization of cell divisome is of great importance to cell physiology and survivability. In our work, we discovered most cell deaths correlated with failure in forming a matured divisome. Geometrical constrictions may lead to non-negligible impact on the positioning of divisome (supplementary Fig. S10) and essential dynamic processes related to cell division, such as nucleoid occlusion^37,38^ and MreB localization^39,40^. The FtsZ-ZapA-ZapB-MatP linkage may play a role in coordinating division with nucleoid segregation^41^. Whether the change of cell width could influence the distance of successive replisomes and the number of replisomes^42^? The Min oscillation system is also reported with a timing effect on cell division^43^. How do the interactions between those concurrent processes affect the temporal scale of cell division when cell width is variable^44^? Those questions are yet to be explored.

## Methods

### Strains and growth medium

All the strains used in this study were *E. coli* K-12 MC4100, constructed, sequenced, and extensively tested by Ariel B. Lindner’s laboratory. Detailed information on strain genotype is included in Table S1. The FtsZ-gfpmut2 expression strain used in the steady-state inhibition experiment is based on a strain with a plasmid pJC85::pBAD42 carrying an extra copy of FtsZ under *P*_lac_ promoter. *E. coli* growth experiments were performed in the LB medium. 25 mg/ml Kanamycin was used to grow the strains with respective resistance markers. The expression of FtsZ-gfpmut2 protein was induced by 50 ug/ml IPTG. Images were shot when cells were grown in LB solid medium (5% agarose) supplemented with 12 μM A22. Detailed information about the medium component is included in Supplementary Table S2.

### Micro-channel PDMS chip preparation

We developed a micro-channel chip that consisted of a thin polydimethylsiloxane (PDMS) layer, an agarose gel layer and two glass coverslips (CITPGLAS. 24 $\times$ 50 mm, thickness 0.13–0.16 mm). PDMS microchannel was prepared by replicating structures from a silicon mold, which contained silicon relief structures with a micron-feature size fabricated using electron beam lithography and dry etching techniques with 100 nm spatial resolution. The silicon mold was treated with the vapor of trimethylchlorosilane for over 15 min before use to prevent the adhesion of PDMS and silicon mold. PDMS oligomers (184 silicone elastomer bases, Sylgard, USA) and cross-linking agent (184 silicone elastomer curing agent, Sylgard, USA) were mixed at a 10:1 mass ratio to prepare PDMS pre-polymer solution. The solution was first pumped to a rough vacuum in a vacuum bell jar to remove air bubbles. Then a droplet of mixed PDMS was poured onto the silicon mold. Thereafter we used a spin-coater to make a PDMS thin film with a spin program: in the first pulse at 500 rpm for 30 s and in the second pulse at 1400 rpm for 10 s Then the PDMS film was cured for about 2 h at 75 °C in an oven (Thermo Scientific, Hera Thermo), which resulted in a thin layer of PDMS film. Then the PDMS film was carefully peeled away from the mold, transferred to a glass coverslip (see Supplementary Fig. S10 for the SEM image of the micro-channels with “necks”). The coverslip was ultrasonically cleaned in ethanol for 10 min and dried by N_2_ gas. To clean the surface of the PDMS film and enhance the surface hydrophilicity, we applied an oxygen plasma treatment for one minute. A droplet of *E. coli* cell suspension was applied onto the PDMS film. To confine the cells and to provide the nutrition to the bacterial cells, a prepared agarose pad was covered on it containing 50 ug/ml IPTG, 12 μM A22. Then we put another glass coverslip onto the top of the agarose pad and used solid paraffin wax to seal the gap between the coverslips. Cells were confined to the PDMS micro-chamber and grown at 30 °C on a temperature-controlled microscope stage for 3 h.

### Cell preparation

Before time-lapse imaging, cells were picked from a single colony on an agar plate which was streaked no more than 7 days before use. The cells were inoculated into 4 mL LB liquid medium with carefully selected antibiotics. After shaking for 12 h at 37 °C in a thermo-state shaker, cells were diluted 100 folds into 2 mL of fresh liquid medium. Incubate again in a shaker at 37 °C till OD_600_ = 0.1–0.4. 1 µl of the bacterial culture liquid was then pipetted onto the PDMS chips on a coverslip. The droplet was then immediately covered with a 5% agarose pad containing LB, 12 µM A22 and 50 ug/ml IPTG.

### Microscopy and image acquisition

We performed simultaneous phase-contrast imaging and epifluorescence imaging on an inverted fluorescence microscope (Nikon Ti-E) with Perfect Focus (TI-PFS-CON2), 100 × oil immersion objective (Nikon plan Apo VC, numerical aperture = 1.4). The microscope was enclosed by a custom-made chamber that was pre-heated and kept at 30 °C. Fluorescence was excited by a lamp (Nikon C-HGFIE) through a neutral density filter. For excitation of GFP signal, cells were illuminated through a GFP filter cube (λ_ex_/λ_bs_/λ_em_ = 450–490/495/500–550 nm). The fluorescence signal was recorded by a Cool SNAP HQ2 camera. The FtsZ dynamics was captured with a time-interval of 2 min. Exposure time for GFP and TXRED florescence both was 3 s, and the bright field was 0.5 s. ND was set as 1/8. Images were recorded by software named ‘MetaMorph’. In our experiment, limited by the size of micro-channels and the scope of microscopic imaging, we can only follow the growth and division of cells for about 3 h, after which over 80% of the cells grow out of the channel or the scope of imaging. Therefore, the results we discussed are based on the experimental data within the first two hours after cell loading and 1 h adaptation.Given we perform all the experiments strictly with same conditions, such as temperature, A22 concentration, cell pre-culture and adaptation time, etc., and the only difference between the cells is the geometric constraints such as the channel width and neck width, it is reasonable to attribute the variation of cell size and their divergence to the difference of geometric constraints but not to the other experimental conditions.

### Image processing

Image processing was performed using FIJI software. Toolboxes were used for image analysis, such as contrast and brightness adjustments. Phase-contrast images and fluorescent images of FtsZ dynamics were adjusted for contrast. When necessary, images were background-corrected using a rolling ball with radius 50 pixels, and median-filtered for 2 pixels with merged fluorescent images. Images were background-subtracted for viewing purposes. Unaltered images were used for quantitative processing in all cases.

### Statistical analysis

The quantitative data are expressed as Mean ± SD. Comparisons between two or more specific sets of data are analyzed by using One-way analysis of variance (ANOVA) test. For all data analyses, the *P* value of < 0.05 are considered statistically significant, and *P* value of > 0.05 are considered no statistically significant.

## Supplementary information

Supplementary Information.

## Data Availability

The data generated during the current study is included in this article and its supplementary files.
