# Supplementary material for: Artificial modulation of cell width significantly affects the division time of Escherichia coli
Source: Sci Rep. 2020 Oct 20;10:17847. doi: 10.1038/s41598-020-74778-3 (PMC7576201; doi:10.1038/s41598-020-74778-3)
Supplement: Supplementary file 1 — Supplementary Information. [file 41598_2020_74778_MOESM1_ESM.docx]

# Supplemental Information

**Artificial modulation of cell width significantly affects the division time of *Escherichia coli***

Baihui Liang, Baogang Quan, Junjie Li, Chantal Loton, Marie-Florence Bredeche, Ariel B.Lindner, and Luping Xu

Contents

[Supplemental figures 1](#_Toc48050159)

[Supplemental tables 7](#_Toc48050160)

[References 10](#_Toc48050161)

# Supplemental figures


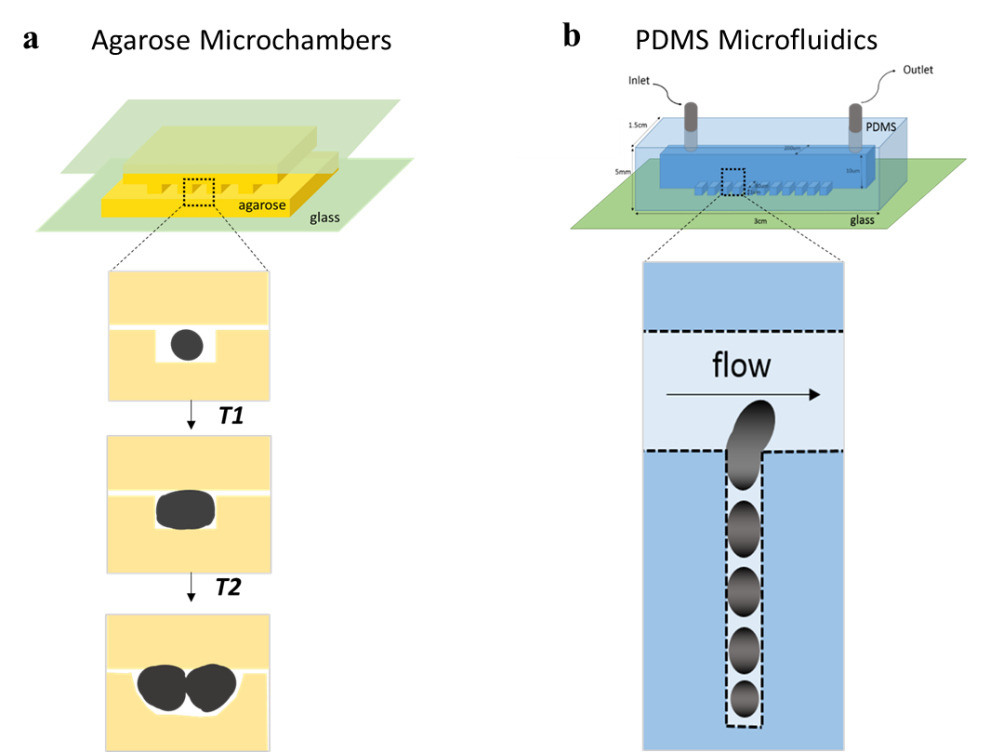


**Figure S1.** Microchambers and microfluidics. **(a)** For microchamber devices, it has disadvantages that agarose would deform when cells have large deformation and it is hard to obtain high-quality images due to the accumulated cell growth^1,2^. **(b)** For microfluidics devices, deformed cells will block the entrance of the channel which will lead to heterogeneous growth within a channel^3^.


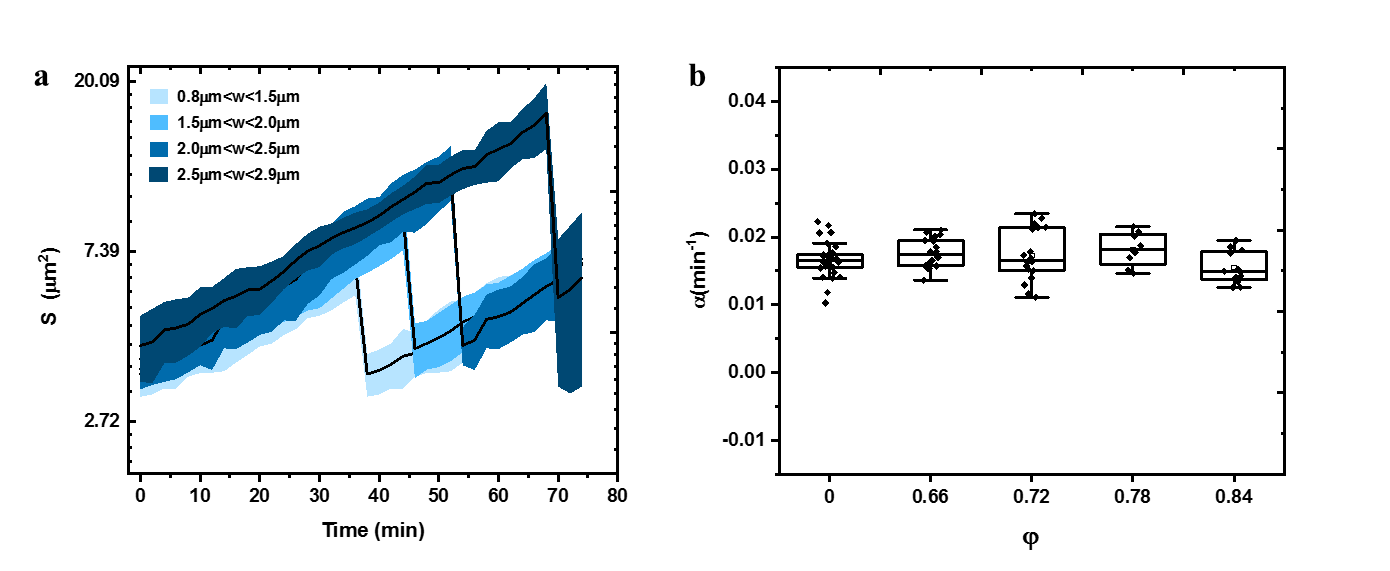


**Figure S2.** Growth rate distribution for cells growing in straight channels and ‘necked’ channels. **(a)** In straight channels, cells were classified into four groups based on their widths. Bold line represents the average cell area (in logarithmic scale) and shades along the bold line represent the standard deviation of the cell area. **(b)** In ‘necked’ channels, cells share a constant growth rate of biomass regardless of their constriction ratio $\varphi$.


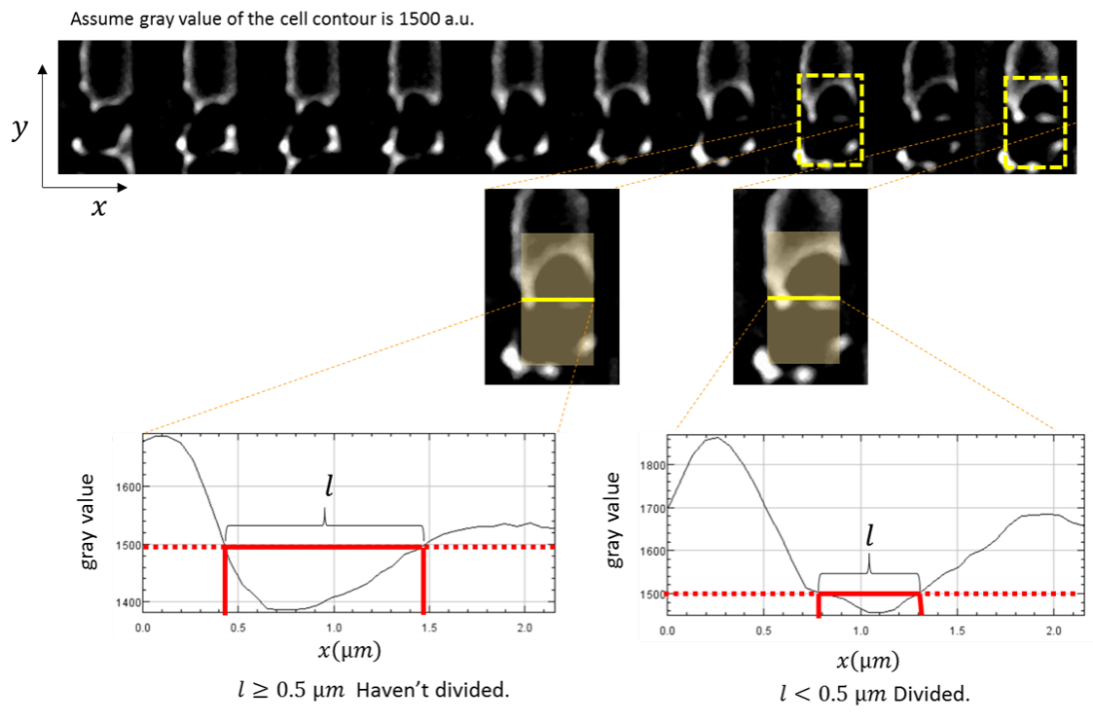


**Figure S3.** The scheme of image analysis for the determination of the end of cell division of a cell. First, we identify the gray value of cell boundary in an image, for instance, 1500 a.u. in this scheme. A pixel with a gray value below 1500 a.u. is recognized as in the cell; otherwise it is on the outside. Next, we measured the width of cell (*l*) at the division point. A cell is recognized as reaching the end of cell division when the distance *l* gets to be less than 0.5$\mu$m (calibrated with fluorescence labeled FtsZ, Data not shown).


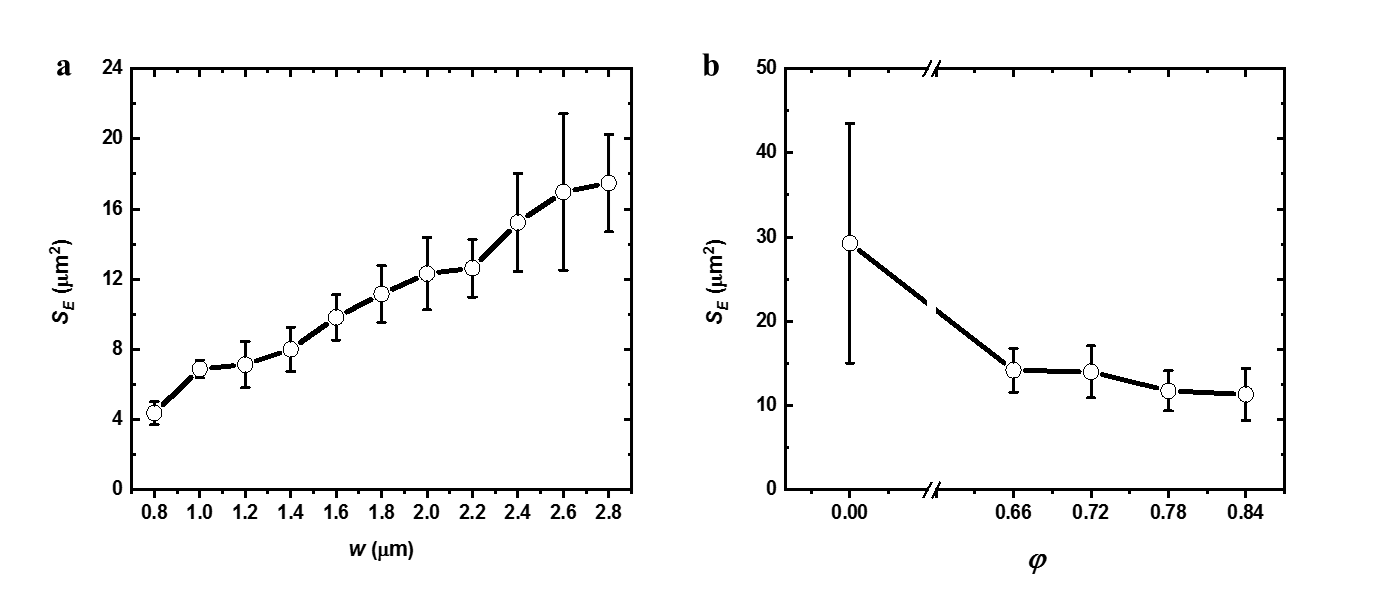


**Figure S4.** Cell area at the end of division (*S_E_*) increases as cell width increases. **(a)** *S_E_* in straight channels increases as cell width increases. **(b)** *S_E_* in ‘necked’ channel is much smaller than that in straight channels.


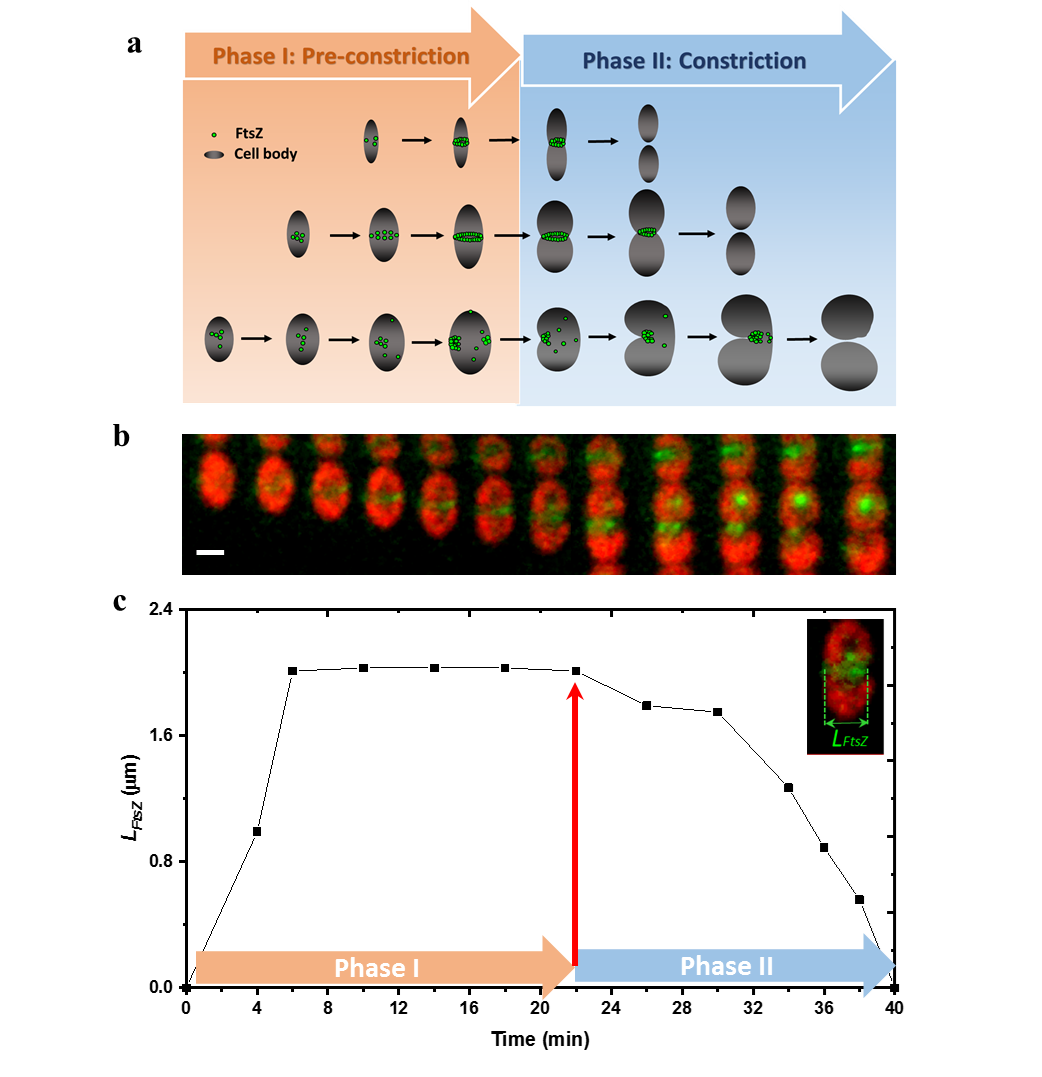


**Figure S5. (a)** Schematic of diverse dynamics of FtsZ clusters inside cells of different widths during the cell division process. **(b)** Multi-fluorescence labeling time-lapse images of a cell grew and divided in the microchannel. Nucleoid HU-mCherry (red), FtsZ-mut2gfp (green). (Scale bar, 2 μm). The time interval of the image sequence is 4 min. **(c)** The length of FtsZ clusters (*LFtsZ*) changes during cell division process. The red arrow refers to the transfer moment from phase I to phase II. The insert is the scheme of the length of FtsZ clusters (*LFtsZ*).


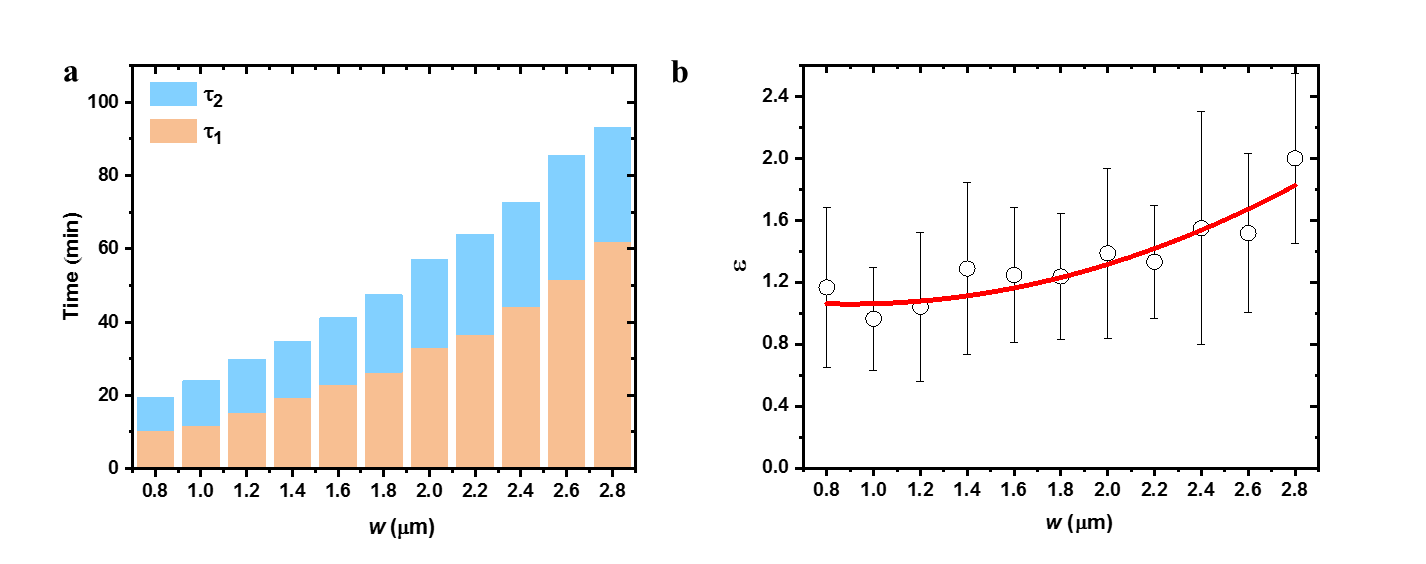


**Figure S6.** The ratio of $\tau_{1}$ and $\tau_{2}$ increases as cell width increases. The solid red line represents exponential fitting (*R^2^*=0.81).


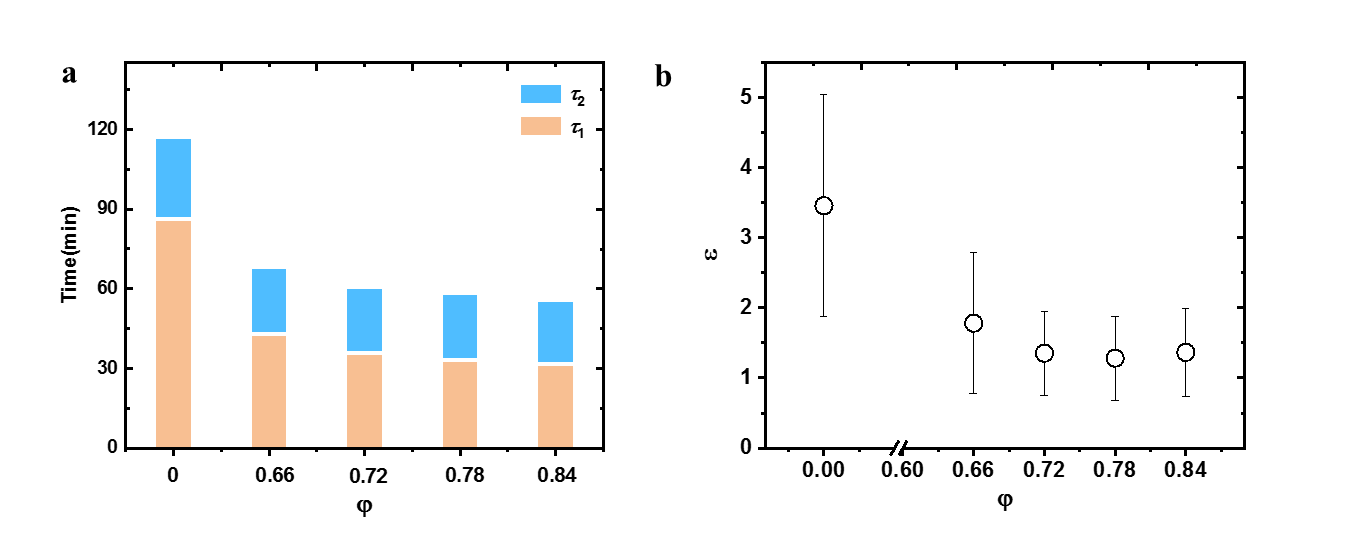


**Figure S7.** The ratio of $\tau_{1}$ and $\tau_{2}$ decreases rapidly as local constriction increases.


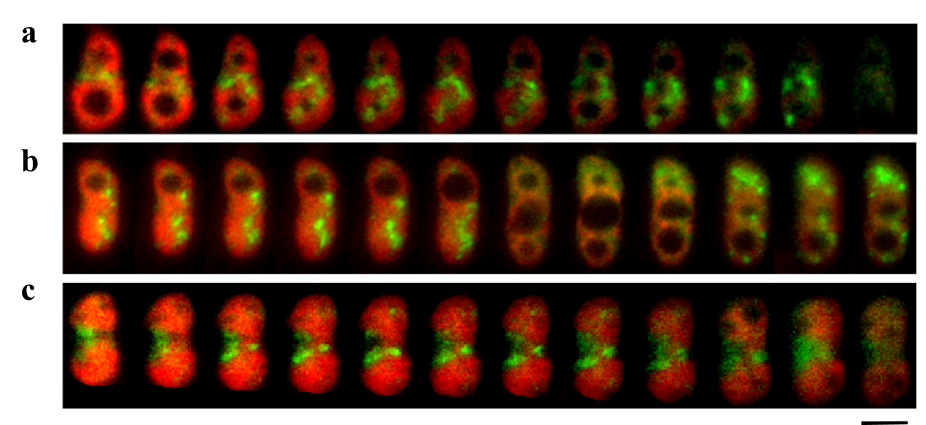


**Figure S8.** **(a)-(c)** represent three examples of the dead cells with multi-fluorescence labeling. while wider cells appear different degrees of damage. Cell was labeled with multi-fluorescent to visualize the death. nucleoid HU-mCherry (red), FtsZ-mut2gfp (green). Cell nucleoid gradually dissipated and damaged severely after A22 treatment for 3 hours. Most cells die of the divisome assemble failure, FtsZ cannot assemble into ring or arc shape structure, and severe cell nucleoid damage indeed results in the failure divisome assemble, which imply the nucleoid-occlusion mechanism^4,5^. The time intervals for the image sequences are 4 min. (Scale bar, 4 μm)


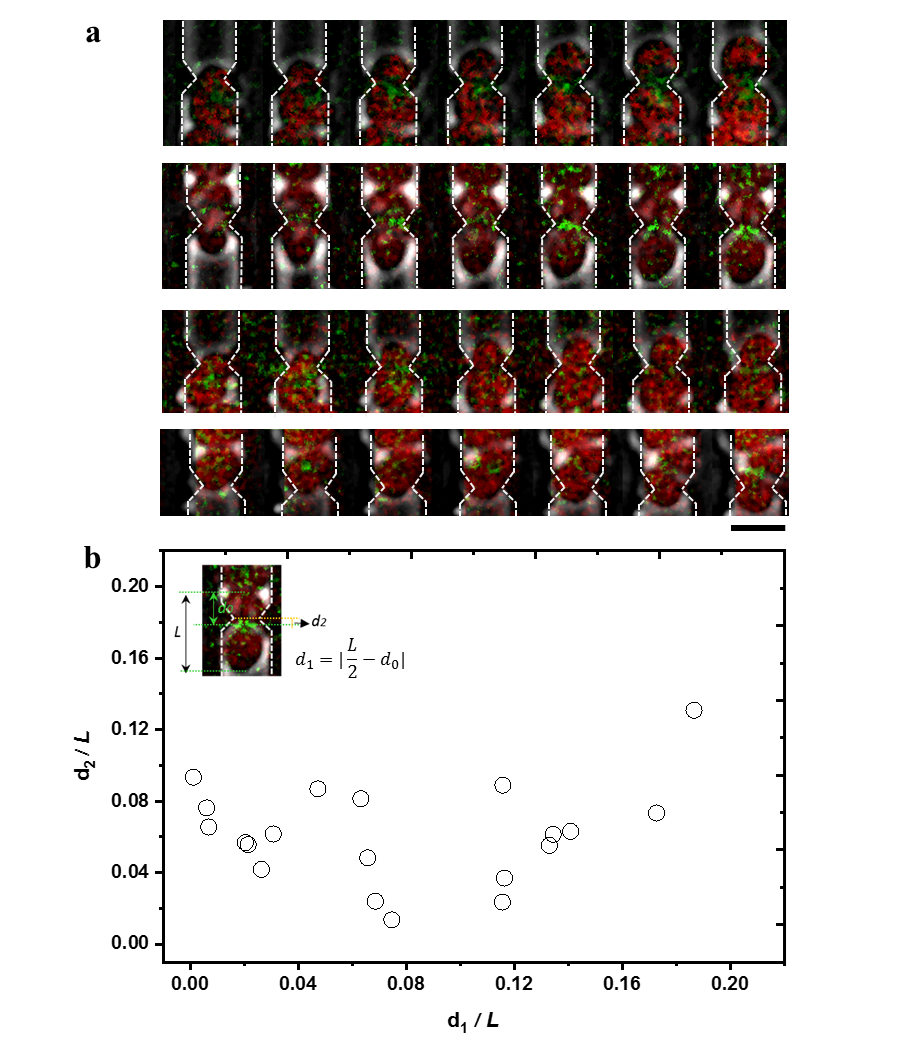


**Figure S9. (a)** Cell division in the ‘necked’ channels, labeled with multi-fluorescence (red: nucleoid HU-mCherry, green: FtsZ-mut2gfp) . Gray dash line represents the border of the ‘necked’ channels. The time intervals for the image sequences are 4 min. (Scale bar, 3.4 μm). **(b)** The impact of neck on the localization of division plane. *d_1_* refers to the distance between the division plane and the middle point along the long axis of the cell, and *d_2_* refers to the distance between the division plane and the position of the neck. L is the length of cell. The result indicates that the cell division plane is biased towards the location of the neck. The insert is the scheme of a representative cell divided in ‘necked’ channel*.*


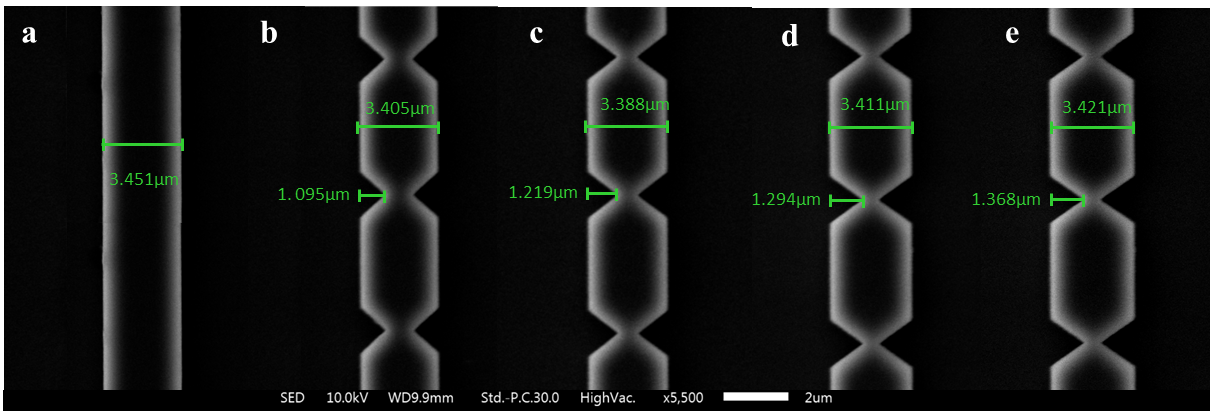


**Figure S10.** The SEM images of the ‘necked’ microchannels without cells. **(a)** represents the straight channel. **(b)-(e)** represent the ‘necked’ channels. The fabrication precision is 100nm.

# Supplemental tables

| **Strain** | **Genotype** | **Note** |
| --- | --- | --- |
| JOE70 | JOE69-lamda::Plac ftsz-gfpmut2, Amp r  Hu-mCherry | This work |

**Table S1**. Strain information.

| **Medium** | **Component** | **Concentration** | **Source** | **Identifier** |
| --- | --- | --- | --- | --- |
| Liquid Medium | LB | N/A | Solarbio | Cat#L1010 |
|  | Kanamycin | 25 ug/ml | Amresco | VT0236 |
| Solid Medium | LB | N/A | Solarbio | Cat#L1010 |
|  | Agarose | 5% w/v | BIOWEST. Spain | Lot.111860 |
|  | IPTG. | 0.1 mM | Solarbio | Cat.No.I1020 |
|  | A22 | 12 μM | Sigma Life Science | CAS 22816-60-0 |

**Table S2.** Medium source information.

| **Cell width**  **(μm)** | **Samples of success division** | **Average division time (min)** | **σ^2^ of division time (min)** |
| --- | --- | --- | --- |
| 0.8 | 20 | 19.5 | 1.512 |
| 1.0 | 10 | 22.5 | 3.375 |
| 1.2 | 40 | 29.9 | 5.242 |
| 1.4 | 66 | 35.12 | 6.629 |
| 1.6 | 79 | 42.37 | 7.629 |
| 1.8 | 93 | 47.23 | 7.740 |
| 2.0 | 56 | 55.79 | 7.372 |
| 2.2 | 32 | 62.69 | 9.539 |
| 2.4 | 15 | 72.93 | 10.250 |
| 2.6 | 5 | 80.4 | 14.724 |
| 2.8 | 5 | 93 | 12.728 |

**Table S3.** Division time related parameters: sample size for each width, average division time and the σ^2^ of the division time. The sample size represents the collected number of cells who constricted in the micro-channel with same width and could be successfully complete the whole cell division cycle.

| $\boldsymbol{w}$ | $\boldsymbol{N}$ | $\boldsymbol{m}$ |
| --- | --- | --- |
| 0.8 | 20 | 0 |
| 1 | 21 | 0 |
| 1.2 | 78 | 0 |
| 1.4 | 66 | 0 |
| 1.6 | 82 | 1 |
| 1.8 | 103 | 3 |
| 2 | 72 | 3 |
| 2.2 | 50 | 4 |
| 2.4 | 48 | 4 |
| 2.6 | 39 | 4 |
| 2.8 | 19 | 2 |

**Table S4.** Parameters of death rate *θ=m/NT* for straight channel. *N* refers total number of the cells in the channel. *m* refers the number of dead cells in the observation time *T.*

| $\boldsymbol{\Delta}$ | $\boldsymbol{\varphi}$ | $\boldsymbol{N}$ | $\boldsymbol{m}$ |
| --- | --- | --- | --- |
| 0 | 0 | 189 | 21 |
| 1.1 | 0.66 | 189 | 18 |
| 1.2 | 0.72 | 190 | 13 |
| 1.3 | 0.78 | 163 | 8 |
| 1.4 | 0.84 | 170 | 2 |

**Table S5.** Parameters of death rate *θ=m/NT* for ‘necked’ channel. *N* refers total cell number during the observation time *T*. *m* refers the death cells number.

|  | Straight channel | ‘necked’ channel |
| --- | --- | --- |
| Function | $ln\theta=a+b\times ln\delta$ | |
| $a$ | -4.08582 ± 0.13038 | -2.70729 ± 0.48872 |
| $b$ | -3.57735 ± 0.47897 | -14.83587 ± 5.48607 |
| Residual Sum of Squares | 0.58154 | 0.5561 |
| Pearson's r | -0.95799 | -0.88614 |
| R-Square (COD) | 0.91774 | 0.78525 |
| Adj. R-Square | 0.90129 | 0.67787 |

**Table S6.** Linear fitting parameters of the correlation between $ln\theta$ and $ln\delta$.

# References

1 Wu, F., van Schie, B. G., Keymer, J. E. & Dekker, C. Symmetry and scale orient Min protein patterns in shaped bacterial sculptures. *Nat Nanotechnol* **10**, 719-726, doi:10.1038/nnano.2015.126 (2015).

2 Shoji Takeuchi, W. R. D., Douglas B. Weibel, and George M. Whitesides. Controlling the shape of filamentous cells of *Escherichia Coli*. *Nano Letter* **5**, 1819–1823. (2005).

3 Ariel Amir, F. B., Dustin B. McIntosh, David R. Nelson, and Suckjoon Jun. Bending forces plastically deform growing bacterial cell walls. *Proc Natl Acad Sci U S A* **111**, 5773-5783 (2014).

4 Cho, H., McManus, H. R., Dove, S. L. & Bernhardt, T. G. Nucleoid occlusion factor SlmA is a DNA-activated FtsZ polymerization antagonist. *Proc Natl Acad Sci U S A* **108**, 3773-3778, doi:10.1073/pnas.1018674108 (2011).

5 Mannik, J. W., F. Hol, F. J. Bisicchia, P. Sherratt, D. J. Keymer, J. E. Dekker, C. Robustness and accuracy of cell division in *Escherichia coli* in diverse cell shapes. *Proc Natl Acad Sci U S A* **109**, 6957-6962, doi:10.1073/pnas.1120854109 (2012).
